# Immunomodulatory effect of *Moringa oleifera *and *Phyllanthus niruri *extracts on anti-HBV cytokine production by human peripheral blood mononuclear cells

**DOI:** 10.12688/openreseurope.20378.2

**Published:** 2025-10-27

**Authors:** Bright Asare, Philip Selorm Segbefia, Rawdat Awuku-Larbi, Diana Asema Asandem, Theophilus Brenko, Lutterodt Bentum-Ennin, Frank Osei, Doreen Teye-Adjei, Georgina Agyekum, Linda Akuffo, Bethel Kwansa-Bentum, Kwadwo Asamoah Kusi

**Affiliations:** 1 Department of Immunology, Noguchi Memorial Institute for Medical Research, College of Health Sciences, University of Ghana, Legon, Accra, Ghana; 2 Department of Animal Biology and Conservation Science, College of Basic and Applied Sciences, University of Ghana, Legon, Accra, Ghana; 3 Department of Molecular Medicine, School of Medicine and Dentistry, College of Health Sciences, KNUST, Kumasi, Ghana

**Keywords:** Hepatitis, Cytokines, Moringa, Phyllanthus, immunomodulatory

## Abstract

**Background:**

Chronic hepatitis B (HBV) infection is a leading cause of cirrhosis and liver cancer globally. The current approved drugs for chronic HBV management include pegylated interferons and nucleoside analogs but these have limited efficacies and some adverse side effects. There is an urgent need to find safer and more effective antivirals for chronic HBV management. This study aimed to evaluate the *in vitro* immunostimulatory properties of the aqueous and ethanolic leaf extracts of the plants *Moringa oleifera* and *Phyllanthus niruri* on human peripheral blood mononuclear cells (PBMCs) from chronic HBV-infected persons yet to commence therapy and HBV-negative persons.

**Methods:**

Plant extracts were freeze-dried and stock solutions prepared for phytochemical analysis. Extracts were used to stimulate cultured PBMCs from HBV-infected and HBV-negative persons and the levels of selected cytokines in culture supernatants measured by a multiplexed Luminex assay. The MTT assay was used to assess extract cytotoxicity.

**Results:**

Aqueous and ethanol extracts of both plants were not cytotoxic but rather increased cell metabolic activity. The extracts induced the release of IL-6, IL-1β, IFN-γ, IL-10, TNF-α and IFN-α in PBMCs from both healthy individuals and chronic HBV patients, but cytokine levels were in most instances significantly higher in PBMCs from healthy individuals compared to HBV infected persons and may be related to the reduced immune responsiveness associated with chronic HBV infection. The reduced responsiveness of immune cells from chronic HBV-infected persons to stimulation may explain viral persistence and development of the chronic state. Overall, leaf extracts from both plants were safe and stimulated the release of HBV replication-limiting cytokines and may be important for chronic HBV management.

**Conclusions:**

These findings lay the foundation for the potential integration of these extracts into HBV management strategies and provide promising data for future therapeutic development aimed at improving immune responses in chronic HBV patients.

## Introduction

Hepatitis B Virus (HBV) is an enveloped virus with a partial double-stranded circular DNA genome that belongs to the *Hepadnaviridae* family. The virus attacks the liver and causes both acute and chronic HBV infections. The virus is mostly transmitted vertically from mothers to children at birth but also through contact with blood or other bodily fluids from an infected person such as through sharing of piercing instruments. In 2022, the World Health Organization (WHO) estimated that 254 million individuals had chronic hepatitis B infection, with 1.2 million new infections yearly. Hepatitis B caused an estimated 820 000 deaths in 2022, the majority of which were due to cirrhosis and hepatocellular carcinoma (primary liver cancer) ^1^.

There is a safe and effective HBV vaccine that elicits the right immune responses to prevent infection with HBV. It is safe and can be effectively used as a routine prophylactic ^2^. The vaccine is however not necessary once an individual has been infected. Infection with HBV can either result in an acute infection that resolves within a period of 6 months, or progress to chronic infection, in which case the infected person may have to live with the virus, in most instances for life. At some stages of infection chronicity, treatment to reduce viral replication or other problems may be required.

Since HBV is a noncytopathic virus, the host's immune response against the viral products is regarded to be a source of immunopathology ^3– 5^. During an acute infection, the virus can either be detected and eliminated by the cytotoxic T lymphocytes (CTL) or by non-cytolytic routes, which involve the production of cytokines that mediate the clearance of the virus ^6^. Cytokines have been found to regulate viral replication and aid in the cure of HBV by targeting different stages of the life cycle of the virus ^7^. Cytokines such as Interleukin (IL)-6, IFN-α, and IL-1β have been found to prevent the entry of the virus into the hepatocytes ^8, 9^. Some of these cytokines also prevent transcriptional activity of the virus thereby inhibiting the replication of the virus. Interleukin-6, IL-1β, IFN-α, TNF-α, and IFN-γ have all been found to inhibit the transcriptional activity of the virus and thereby inhibit viral replication ^10– 13^

However, when the immune system is unable to get rid of the virus, the infection progresses to chronic forms, characterized by complications such as cirrhosis, hepatocellular cancer, and liver failure. Preventing the progression of the disease to these complicated forms is the major therapeutic goal for persons with chronic HBV infection. For these patients, there are two available forms of treatment; either short-term treatments with immunomodulators like standard or pegylated interferon, or long-term treatments with nucleoside analogues like adefovir, dipivoxil, tenofovir, lamivudine, entecavir, or telbivudine ^14^. These medications prevent or limit viral replication and reduce liver inflammation and damage. IFN-α directly inhibits HBV replication in hepatocytes and indirectly reduces hepatitis B viral load by modulating the host immune response, whereas nucleoside analogs suppress the replication of the virus by targeting viral reverse transcriptase ^15, 16^. However, these drugs have been found to have limited efficacies and some undesirable side effects and are associated with the emergence of anti-viral drug resistance ^17^, highlighting the need for affordable and readily available alternatives.

Medicinal plants have been utilized to cure various human ailments, including many infectious diseases ^18^. As a result, further research into antiviral compounds produced from medicinal plants is an appealing strategy for developing new anti-HBV drug candidates. Chronic liver disease, particularly chronic hepatitis B infection, has long been treated with preparations from the plant *Phyllanthus niruri* ^19, 20^. Thyagarajan and his team postulated that treatment with a decoction of *P. niruri* may inhibit HBV DNA replication and result in seroconversion of HBsAg ^21^. *Phyllanthus niruri* has various pharmacological properties, including antibacterial, immunomodulation, antiviral, anti-hyperglycemia, diuretic, and hepatoprotective activities, and could be used particularly for treating HBV infections ^22^. *Moringa oleifera* extracts have also been found to contain components with antioxidant, antiviral, and anti-fibrotic properties. *M. oleifera* is said to have hepatoprotective and immunomodulation activities and has been used for the management of HBV ^23, 24^. Preparations from this plant have been shown to lower HBV covalently closed circular (ccc)DNA levels and contain compounds that may inhibit HBV replication ^25^.

The observed characteristics of these plants are usually attributed to their rich phytochemical composition, such as flavonoids, tannins, alkaloids, and saponins. According to recent studies, these phytochemicals may control the production of cytokines by influencing immunological pathways ^26^. Furthermore, it has been demonstrated that alkaloids and flavonoids improve JAK-STAT signaling, which is critical to regulating interferons (IFN-α and IFN-γ), cytokines necessary for antiviral defense ^26^. Saponins and polyphenols may influence the mitogen-activated protein kinase (MAPK) pathway, which is crucial for immune cell activation, to promote cytokine release and immune modulation ^26^. *Phyllanthus niruri* and Moringa oleifera are household plants in Ghana that are traditionally used to treat numerous diseases. They were selected for this study based on their ethnobotanical relevance, their known immunomodulatory properties, and emerging evidence of their direct anti-HBV effects. The main aim of this study was to evaluate the ability of *M. oleifera* and *P. niruri* crude leaf extracts to stimulate the non-cytolytic pathway of immune cells as this pathway is believed to limit the replication of HBV.

## Methods

### Ethics statement

This study is part of a larger ongoing study titled “ *Clinical and Immunopathological Consequences of Hepatitis B and Malaria Co-infections* (HEPMAL), based at the Noguchi Memorial Institute for Medical Research (NMIMR), University of Ghana. The study received scientific approval from the Scientific and Technical Committee, NMIMR. Ethical approval was obtained from both the Institutional Review Board (IRB, Federalwide Assurance number FWAA00001824) of NMIMR (NMIMR-IRB/046/19-20, original approval date: 8 ^th^ January 2020) and the Ethics Review Committee of the Korle-Bu Teaching Hospital, Accra (KBTH-IRB/00024/2020, original approval date: 27 ^th^ May 2020). Archived cells used in this study were obtained from study participants who gave written informed consent and willingly agreed to be part of the study. All experiments were conducted following the relevant guidelines and regulations, including the principles of the Belmont Report and the Declaration of Helsinki.

### Collection and identification of plant extracts

The fresh leaves of *M. oleifera* Lam and *P. niruri* Linn were collected from the University of Ghana's main campus. These were identified at the herbarium of the Centre for Plant Medicine Research (CPMR) at Akuapem Mampong, where voucher specimens with numbers CPMR 5076 ( *Moringa oleifera* Lam) and CPMR 5118 ( *Phyllantus niruri* Linn) have been deposited. The collection of plant material and the experiments conducted with the processed materials followed the national guidelines of Ghana.

### Preparation of extracts

In Ghanaian households, traditional preparations of medicinal plants like *M. oleifera* and *P. niruri* mostly involve boiling in water (aqueous extract) and steeping in local spirits (ethanolic extract). We aimed to scientifically validate these traditional practices. Our goal was to determine if these commonly prepared and consumed extracts exhibit safe and beneficial immunomodulatory effects, hence the use of solvents that are similar to the traditionally used solvents for extraction. The leaves of both plants were washed and shade-dried for three (3) days. The dried leaves of each plant were pulverized into a powdered form. Twenty grams (20 g) of the powdered sample was soaked in 200 ml of distilled water or 70% ethanol. The distilled water-based mixture was boiled to 80 °C for one hour, while the extract that was soaked in 70% ethanol was mixed by shaking and placed in a water bath at 37 °C overnight. The extracts were filtered and partially dried using the rotary evaporator (Julabo, USA). After rotary evaporation, the extracts were transferred into different freeze-drying cups, tightened well under sterile conditions, and kept at -80 °C overnight. They were then lyophilized using the LABCONCO Freezone 4.6 L to preserve the quality and integrity of the extract constituents ^27^.

After the freeze-drying process, the plant extracts were prepared by weighing eight milligrams (8 mg) of each dried powder into 10 ml of distilled water to yield a stock concentration of 800 μg/ml. The stock solution was vortexed well to dissolve the powder and later filtered through a 0.2μm pore filter unit and then stored at -20 °C until use. A two-fold serial dilution was carried out to obtain the working concentrations; 800 μg/ml, 400 μg/ml, 200 μg/ml, 100 μg/ml, 50 μg/ml, and 25 μg/ml. 

### Quantitative determination of phytochemicals in extracts

Quantitative determination of the total amount of phytochemicals in the two extracts was performed as described by 28, with few modifications.

### Determination of alkaloids

A hundred milligrams (100 mg) of the lyophilized samples were mixed with 10 % acetic acid solution in ethanol and kept at 28 °C for 4 hours to create a suspension, which was then further filtered. The filtrate was concentrated to a quarter of its original volume, and then drops of concentrated aqueous NH _4_OH were added to precipitate the alkaloids. The precipitate was then dried in the oven at 80 °C after being rinsed with a 1% ammonia solution. The amount of alkaloid in the sample was determined and represented as mg/g of sample.

### Determination of saponins

Fifty milligrams (50 mg) of the powdered sample were mixed with 5 ml of isobutyl alcohol and stirred. This was followed by the addition of 20 ml of 40 % saturated magnesium carbonate. The resulting solution was then filtered. About 2 ml of 5 % FeCl _3_ solution was added to 1 ml of the colourless filtrate, and the mixture was kept at room temperature for 30 minutes to fully develop the colour. The absorbance of both standard and sample was read at 380 nm with a UV spectrophotometer (Shimadzu, Japan).

### Determination of total phenols

About 300 mg of extract was added to 5 ml of Ammonium hydroxide, followed by the addition of 10 ml of distilled water. The mixture was allowed to stand for 30 minutes to allow for colour to develop. The absorbance of both standard and samples was read at 505 nm with a UV spectrophotometer (Shimadzu, Japan).

### Determination of tannins

To 100 mg of the extract, 10 ml of distilled water was added. The mixture was boiled and filtered, followed by the addition of 5 ml of Folin’s reagent and 10 ml of saturated Na _2_CO _3_. The resulting mixture was left to stand for 30 minutes for the colour to develop. The absorbance was read at 700 nm with a UV spectrophotometer (Shimadzu, Japan).

### Determination of flavonoids

Five milligrams (5 mg) of the extracts were added to 2 M HCl and the mixture was boiled and then filtered after cooling. The filtrate was then diluted with an equal volume of ethyl acetate. The weight of precipitated flavonoids was determined and expressed as mg/g of sample.

### Extract fingerprinting

HPLC fingerprinting was performed on the aqueous and ethanol extracts of *Moringa oleifera* and *Phyllanthus niruri* leaves. A concentration of 10 mg/mL of each extract was prepared in either water or ethanol, vortexed and filtered through 0.20 um Millipore filter. HPLC analysis was performed using an Agilent 1100 system (Santa Clara, CA, USA). Chromatographic separation was carried out on a TSKgel ODS C18 (250 x 4.6 mm i.d., 5 μm particle size) analytical column maintained at 30 ℃. Water in 0.1 % Phosphoric acid (A) and methanol (B) were used as mobile phase at a flow rate of 1 ml/min. The gradient program used was set as follows: 0–10 min, 10–30 %B; 10–20 min, 30–50 %B; 20–30 min, 50–90 %B; 30–35 min, 90–90 %B; 35–38 min, 90–10 %B; 38–40min, 10–10 %B. The column temperature was maintained at 40 ℃ and the injection volume at 20 μl and the wavelength was monitored at 254 nm.

### PBMC isolation and cell stimulation assay

Whole blood was taken from ten (10) patients with chronic HBV infection but have not progressed to the stage where treatment is required, and ten (10) HBV-negative volunteers. All volunteers were participants from the larger HEPMAL study. The isolation of PBMCs was done by differential centrifugation using Ficoll (density = 1.077 g/ml) (Cytiva, Sweden AB), at 1200 x g for 20 minutes at room temperature with the centrifuge brakes off, to prevent the disruption of the density gradient during deceleration (Sun & Sethu, 2018). Cells were then washed in RPMI-1640 (Supplemented with 1% Penn/Strep) at 300 x g for 10 minutes and then placed in RPMI-1640 media (sigma, USA) (Supplemented with 1% non-essential amino acids, 1% L glutamine, 10% FBS, 1% sodium pyruvate, and 1% Penn/Strep). The cells were counted using the Countess II machine ^®^ which relies on the Trypan blue exclusion method.

For the cell stimulation assay, the PBMCs were cultured at a concentration of 2.0 X10 ^5^ cells/ml with the plant extracts at concentrations of 25, 200, and 800 μg/ml. These concentrations were selected to represent the lower, midpoint, and highest concentrations from the MTT assay, described below. The positive control used in the cell stimulation assay was Phytohemagglutinin (PHA) at a concentration of 5 μg/ml. The cultures were kept at 37 °C, 5% CO _2_ in a humidified atmosphere for 48 hours and the supernatant was harvested afterward and stored at -80 °C until use. All cytokine assays were performed with three independent replicates. The inclusion criteria for the archived PBMCs and the MTT assay were that the cell count should not be less than 2.0 × 10 ^5^ cells/ml to ensure sufficient viability and reliability of the assays.

### Luminex multiplex assay

The Luminex multiplex assay was used to evaluate the levels of inflammatory analytes in stored culture supernatants. The Human Premixed Multi-Analyte kit (6-plex panel with IL-10, IL-6, IFN-γ, IFN-α, TNF-α, and IL-1β) was used according to the manufacturer’s instructions (R&D systems, Bio-Techne, USA). These cytokines and inflammatory mediators were selected because of their ability to contribute to limiting HBV replication.

The test used 50 μl of sample to capture an analyte on analyte-specific colour-coded magnetic beads coated with capture antibodies. Fifty microliters (50 μl) of diluted microparticle cocktail were added to each well and the plate was incubated for 2 hours at room temperature on a plate shaker at a speed of 120 x g. The plates were washed three times by the addition of 100 μl/well of the wash buffer. Fifty microliters (50 μl) of diluted biotin-antibody cocktail were added to each well and the plates were incubated for 1 hour on a plate shaker at 120 x g, followed by washing. Fifty microliters (50 μl) of diluted streptavidin-PE were added to each well, and the plate was incubated for 30 minutes on a shaker at 120 x g. Hundred microliters (100 μl) of wash buffer were added to each well and incubated for 2 minutes on the shaker at 120 x g. The plate was read using the Luminex MAGPIX analyzer (Luminex Corporation).

### MTT assay

Cell viability and cytotoxicity were measured using the 3-(4, 5 dimethylthiazol-2-yl)-2, 5 diphenyltetrazolium bromide (MTT) test (Millipore Sigma, USA). The cells were cultured in a 96-well plate with different concentrations (25, 50, 100, 200, 400, 800 μg/ml) of the plant extracts at 37 °C with 5% CO _2_, at a concentration of 2.0 X10 ^5^ cells/ml, for 96 hours. An aliquot of 20 μl of the MTT (5 mg/ml, Sigma, USA) solution was added to each well and the plates were incubated at 37 °C, for 4 hours in a humidified atmosphere with 5% CO _2_. After the insoluble purple formazan crystals are formed by viable cells, DMSO is required to dissolve these crystals completely to allow for colorimetric quantification. Therefore, 100 μl DMSO was added to all wells (including untreated cell controls and extract-treated wells) to dissolve the formazan crystals before measuring the absorbance. Percent Succinate dehydrogenase enzymatic activity (% SDH) for treated cells was calculated for each tested concentration and compared to the enzyme’s activity for untreated cells ^29^. The negative control for this assay was the untreated cells (culture media only).

$$\%\;\text{SDH}\;\text{enzymatic}\;\mathrm{activity}\;=\frac{\left(OD\;test-OD\;control\right)}{OD\;control}x\;100$$

### Data and statistical analyses

All data were checked for normality and appropriate statistical tests were selected for analyses. For the cell viability assay, data were analyzed using a one-way ANOVA to evaluate the effect of different extract concentrations and controls on cell viability. The assumptions of ANOVA, including normality and homogeneity of variances, were tested using the Shapiro-Wilk test and Levene's test, respectively. Post-hoc comparisons were performed with Tukey’s multiple comparison tests to identify significant differences between paired groups while controlling for family-wise error rates. For the Luminex assay data, the Mann-Whitney U test, a non-parametric alternative to the t-test, was employed to compare the cytokine levels between uninfected persons and chronic HBV-infected persons. This method was chosen due to the non-normal distribution of the data, as verified by the Shapiro-Wilk test. GraphPad Prism Version 9.0.0 (GraphPad Software, www.graphpad.com) was used for all statistical analyses and differences were statistically significant when test p-values were less than 0.05.

## Results

### Quantitative phytochemical analysis of *M. oleifera* and *P. niruri* leaf extracts

Following extract preparation, quantitative phytochemical analyses were performed to determine the phytochemical constituents present in leaf extracts from the two plants. The constituent with the highest content per gram of leaves extracted was the flavonoids. The aqueous extract of *P. niruri* had the highest content while the ethanol extract of *M. oleifera* had the lowest flavonoid content ( Table 1). Comparatively, the aqueous extracts of leaves of the two plants had more saponins and alkaloids per gram of leaves extracted. The *P. niruri* aqueous extract also had a much higher content of flavonoids compared to the ethanol extract. Finally, both the aqueous and ethanol extract of *P. niruri* had comparatively higher levels of tannins and phenols compared to the corresponding *M. oleifera* extracts ( Table 1).

**Table 1.** Phytochemical composition of plant extracts.

Phytochemical constituents	*P. niruri* aqueous extract (mg/g)	*P. niruri* ethanol extract (mg/g)	*M. oleifera* aqueous extract (mg/g)	*M. oleifera* ethanol extract (mg/g)
Saponins	5.4	2.6	5.6	3.2
Flavonoids	21.2	3.6	14.8	12.4
Alkaloids	4.6	1.1	3.6	0.4
Tannins	2.3	2.7	1.4	1.2
Phenols	3.1	5.1	1.8	2.0

### HPLC fingerprinting

HPLC fingerprinting was performed on all extracts, and the resulting chromatograms are presented as supplementary figures 1 and 2 (uploaded as extended data to the data repository with DOI 10.17605/OSF.IO/RX73V). Each chromatogram peak represents a distinct phytochemical compound present in the extract. The complex profiles observed confirm the presence of a diverse mixture of compounds in all four extracts. The variation in peak patterns, heights, and retention times between the aqueous and ethanol extracts of the same plant highlights the differential solubility of their constituent phytochemicals. This analysis was for fingerprinting purposes only and the specific identity of the compounds corresponding to each peak was not determined in this study. These chromatograms however provide a foundational reference for future research to isolate, identify, and quantify the specific active immunomodulatory constituents.

### Cytotoxicity and viability of PBMCs upon treatment with plant extracts

**Figure 1.**
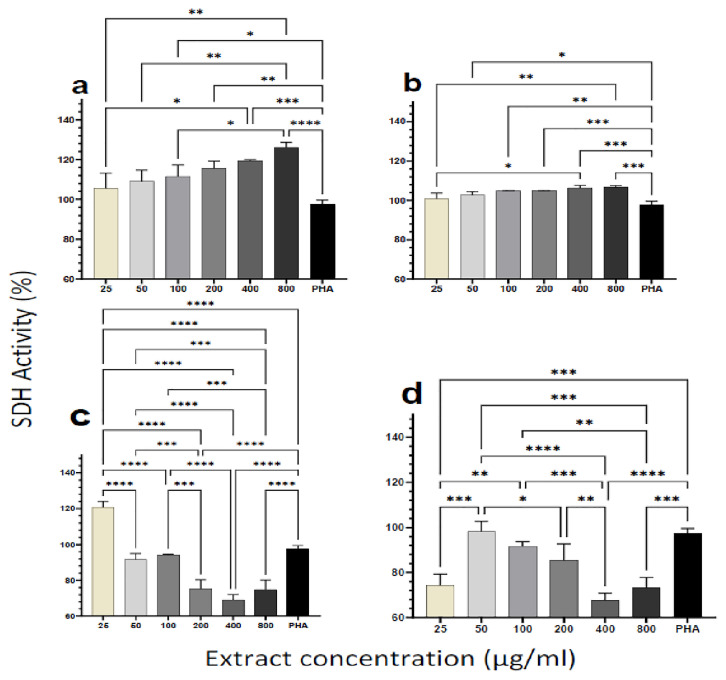
Cytotoxic effect of different concentrations of *M. oleifera* and as *P. niruri* aqueous extracts. Tested extracts include *M. oleifera* aqueous ( **A**) and ethanol ( **C**) extracts as well as *P. niruri* aqueous ( **B**) and ethanol ( **D**) extracts on human PBMCs. Phytohaemagglutinin (PHA) was used as a positive control stimulant. The results presented in each bar are the average of three replicate measurements. Statistical comparisons were performed using one-way ANOVA and Tukey’s multiple comparisons test.

To assess the cytotoxicity effect of plant extracts on the cells, we measured the succinate dehydrogenase (SDH) enzyme activity using a standard MTT assay kit (Millipore Sigma, USA). Human PBMCs from healthy individuals were incubated with increasing doses of the plant extracts (25, 50, 100, 200, 400, 800 μg/ml). Even at relatively high dosages (800 μg/ml), the results indicated that none of the extracts caused cell damage ( Figure 1). For the aqueous extracts from both plants, SDH activity increased significantly with increasing extract concentration and in a dose-dependent manner ( Figures 1A & 1B). For the ethanol extracts of the two plants, however, SDH activity showed a different trend. SDH activity decreased with increasing concentration of the *M. oleifera* ethanol extract ( Figure 1C). A similar observation was made for the ethanol extract of *P. niruri*, except for the lowest concentration of extract (25 μg/ml) yielding similar lower SDH activity as the two highest extract concentrations (400 and 800 μg/ml) ( Figure 1D). Overall, none of the extracts caused measurable cytotoxic effects based on the MTT assay outcome.

Overall, the aqueous extract of *P. niruri* had the best stimulatory activity, with the lowest EC50 among the four extracts (Supplementary Table 1).

### Cell stimulation and luminex multiplex assay

**Figure 2.**
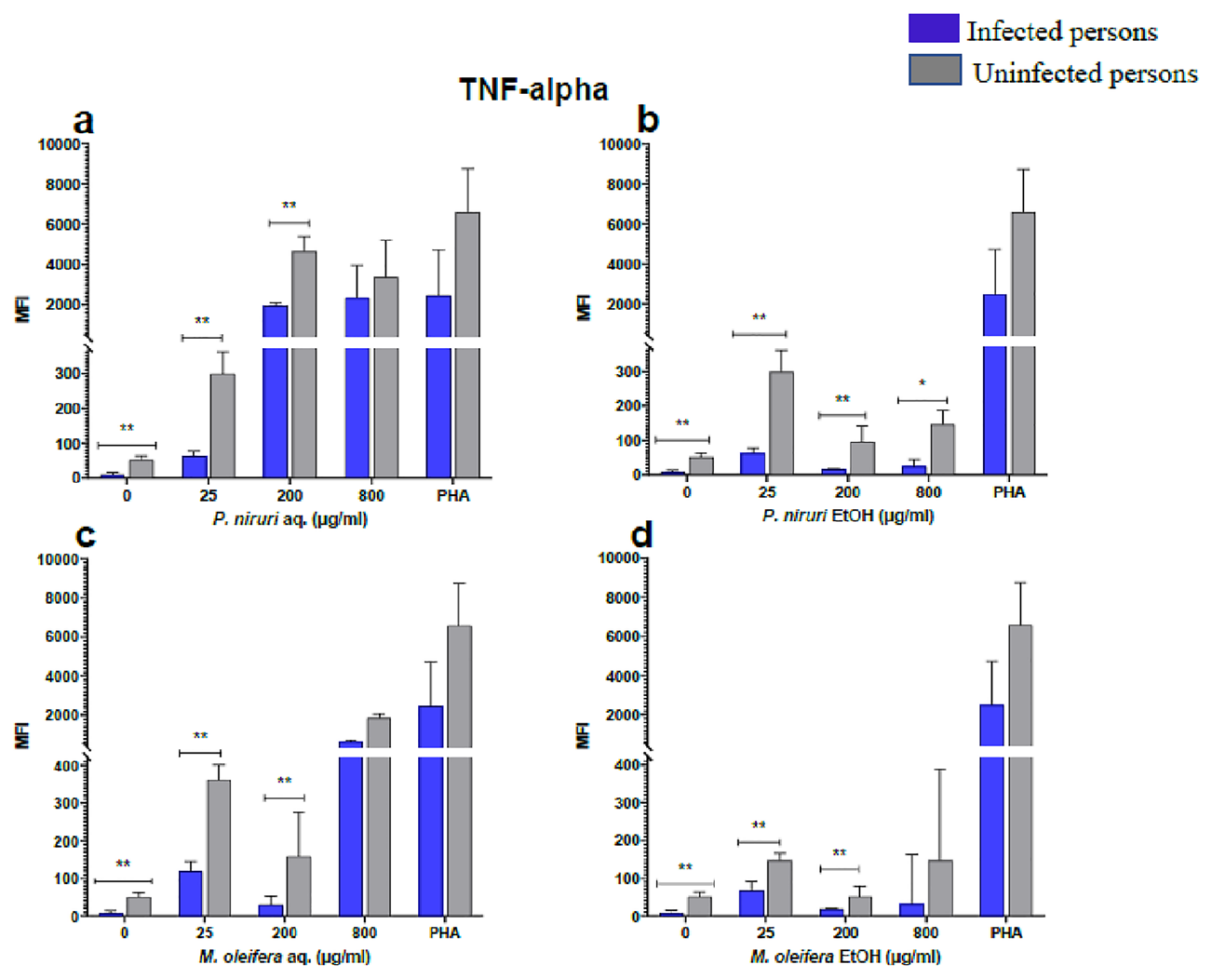
TNF-α levels following stimulation of PBMCs from HBV-positive and uninfected individuals. Stimulation was with aqueous ( **A**) and ethanol ( **B**) extracts of *P. niruri* and aqueous ( **C**) and ethanol ( **D**) extracts of *M. oleifera*. Results presented in each bar are the average of data from 10 individuals. Comparisons between paired samples from HBV-positive persons and uninfected controls were done using the Mann-Whitney U test.

**Figure 3.**
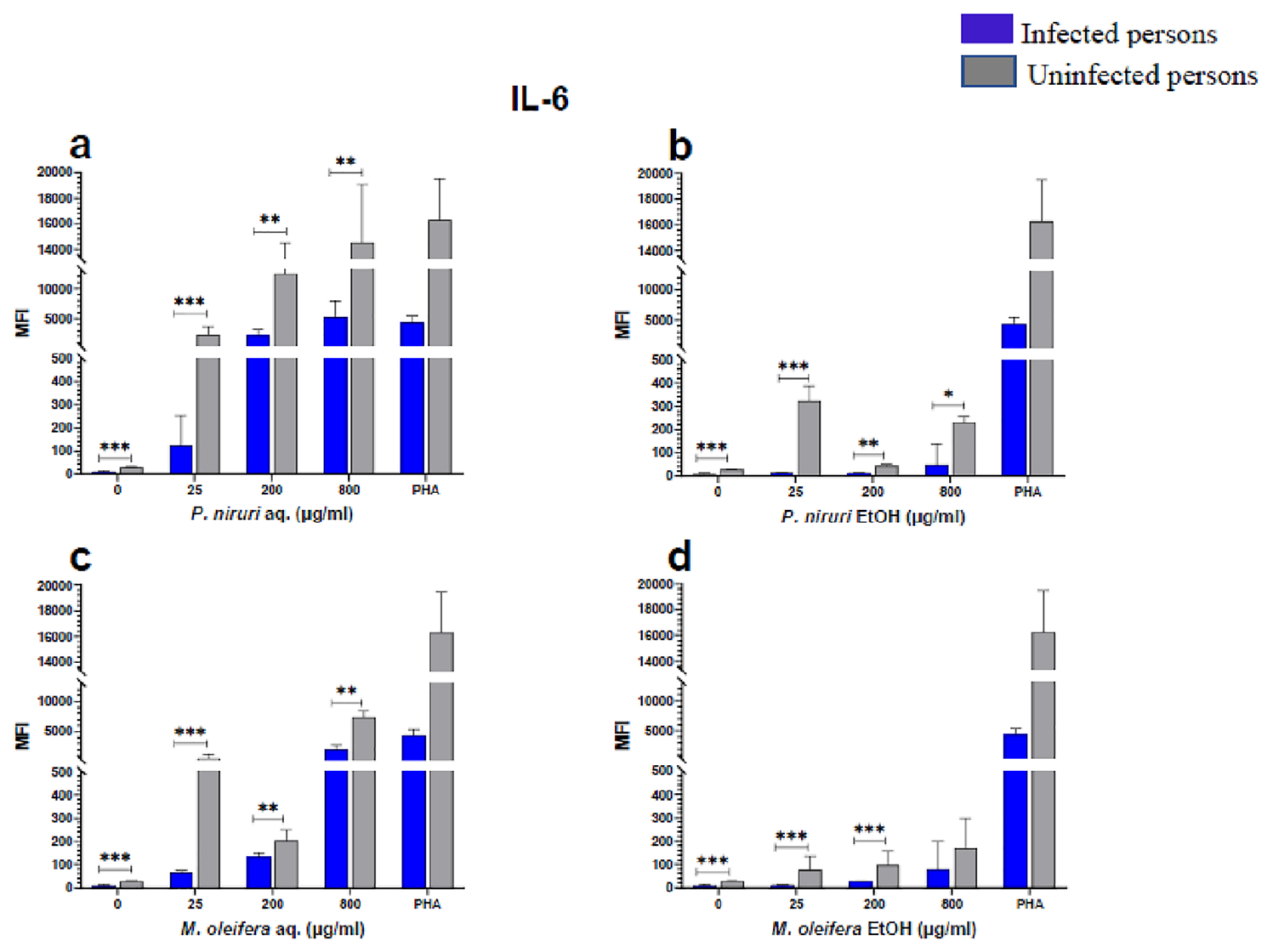
IL-6 levels following stimulation of PBMCs from HBV-positive and uninfected individuals. Stimulation was with aqueous ( **A**) and ethanol ( **B**) extracts of *P. niruri* and aqueous ( **C**) and ethanol ( **D**) extracts of *M. oleifera*. Results presented in each bar are the average of data from 10 individuals. Comparisons between paired samples from HBV-positive persons and uninfected controls were done using the Mann-Whitney U test.

**Figure 4.**
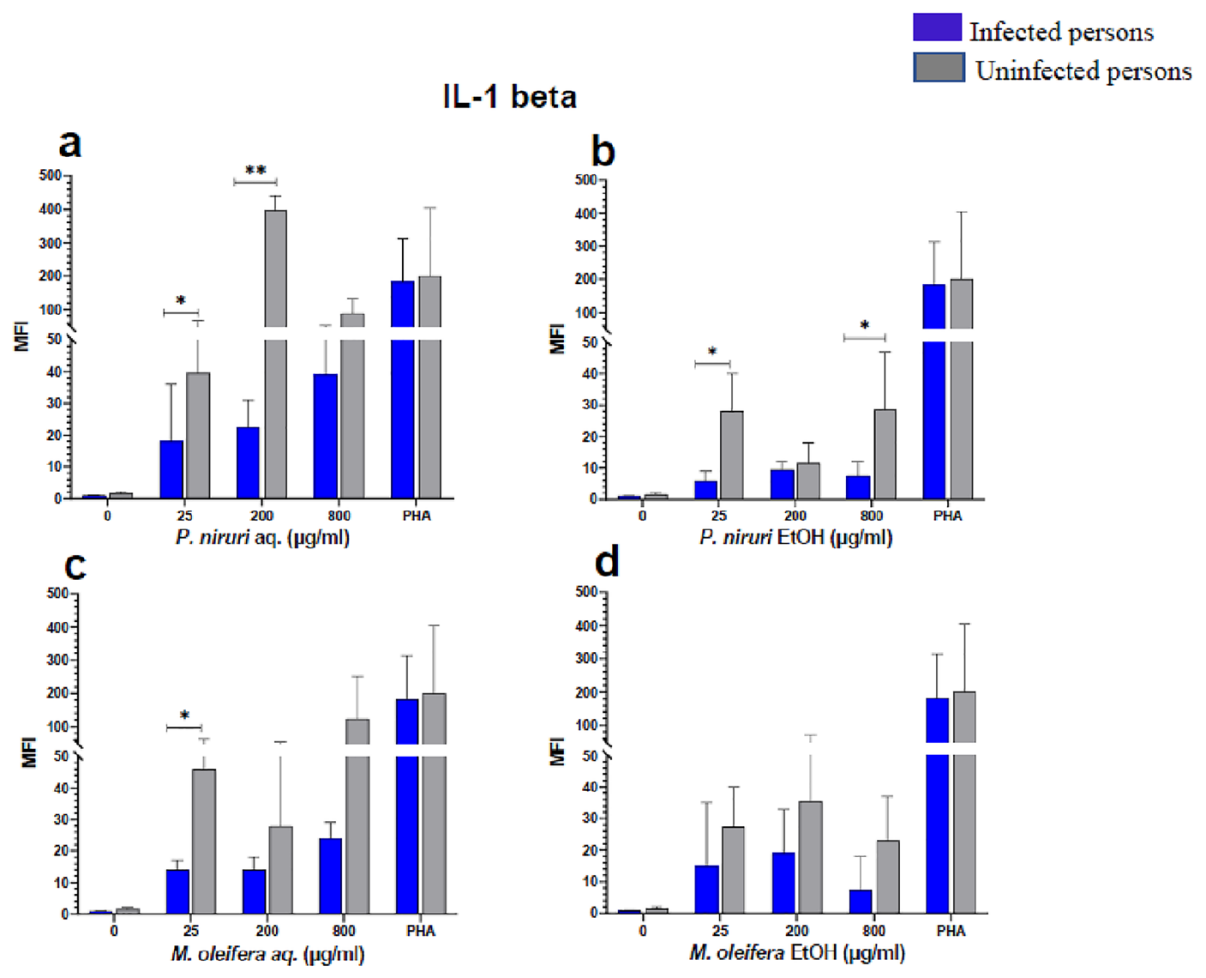
IL-1β levels following stimulation of PBMCs from HBV-positive and uninfected individuals. Stimulation was with aqueous ( **A**) and ethanol ( **B**) extracts of *P. niruri* and aqueous ( **C**) and ethanol ( **D**) extracts of *M. oleifera*. Results presented in each bar are the average of data from 10 individuals. Comparisons between paired samples from HBV-positive persons and uninfected controls were done using the Mann-Whitney U test.

**Figure 5.**
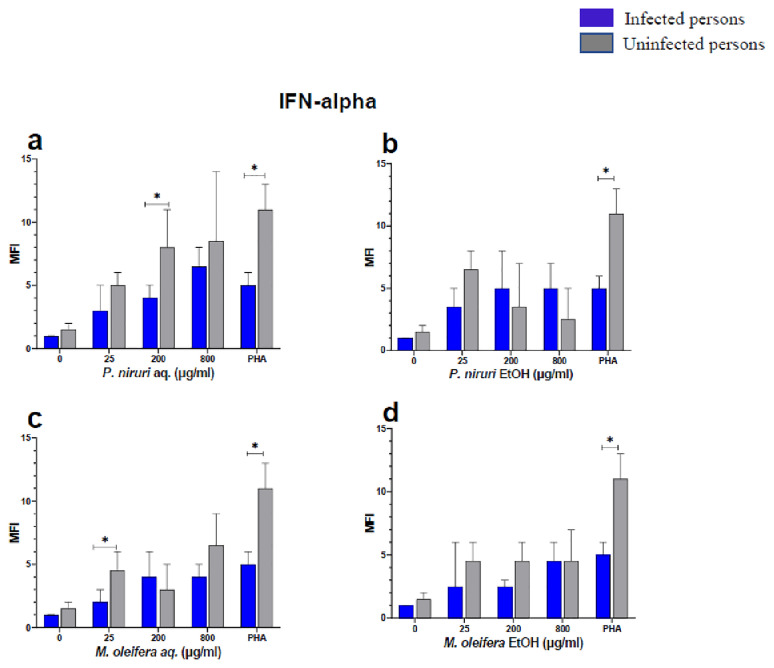
IFN-α levels following stimulation of PBMCs from HBV-positive and uninfected individuals. Stimulation was with aqueous ( **A**) and ethanol ( **B**) extracts of *P. niruri* and aqueous ( **C**) and ethanol ( **D**) extracts of *M. oleifera*. Results presented in each bar are the average of data from 10 individuals. Comparisons between paired samples from HBV-positive persons and uninfected controls were done using the Mann-Whitney U test.

**Figure 6.**
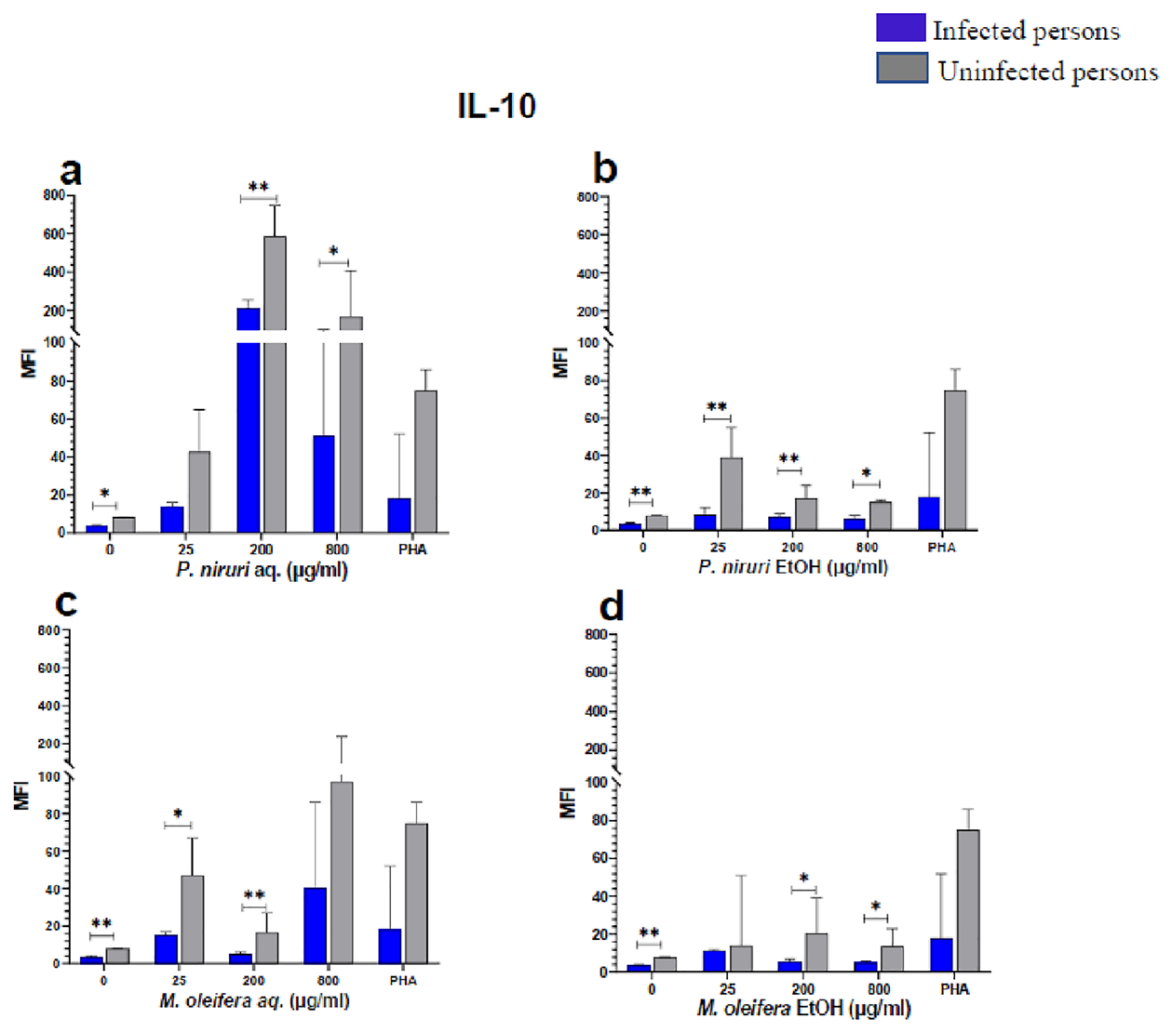
IL-10 levels following stimulation of PBMCs from HBV-positive and uninfected individuals. Stimulation was with aqueous ( **A**) and ethanol ( **B**) extracts of *P. niruri* and aqueous ( **C**) and ethanol ( **D**) extracts of *M. oleifera*. Results presented in each bar are the average of data from 10 individuals. Comparisons between paired samples from HBV-positive persons and uninfected controls were done using the Mann-Whitney U test.

**Figure 7.**
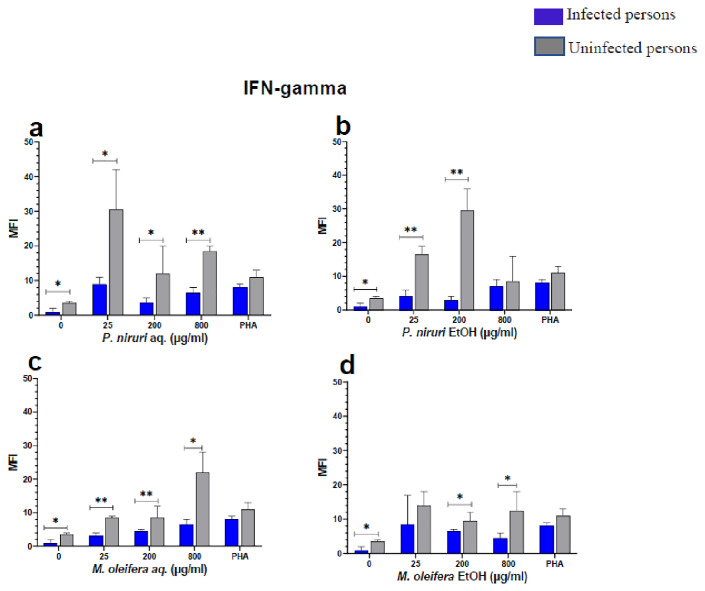
IFN-γ levels following stimulation of PBMCs from HBV-positive and uninfected individuals. Stimulation was with aqueous ( **A**) and ethanol ( **B**) extracts of *P. niruri* and aqueous ( **C**) and ethanol ( **D**) extracts of *M. oleifera*. Results presented in each bar are the average of data from 10 individuals. Comparisons between paired samples from HBV-positive persons and uninfected controls were done using the Mann-Whitney U test.

The effect of the plant extracts on cytokine induction was carried out by culturing PBMCs from chronic HBV patients and HBV-negative controls with the different concentrations of the plant extracts for 48 hours, followed by supernatant cytokine level measurement by the Luminex multiplex assay. Samples were analyzed by measuring the total mean fluorescence intensity (MFI) of the different cytokines, including TNF-α ( Figure 2), IL-6 ( Figure 3), IL-1β ( Figure 4), IFN-α ( Figure 5), IL-10 ( Figure 6), IFN-γ ( Figure 7). For each of these analytes, there were generally higher levels of cytokines produced by PBMCs from HBV-negative individuals compared to the HBV-positive individuals, suggesting the possibility of some impairment of the responsiveness of PBMCs from the HBV-infected persons. As was observed in the MTT assays, there was a general trend of increase in cytokine levels from both HBV-infected and uninfected persons with extract concentration when the aqueous extracts were used for stimulation. For ethanol fractions, however, there is no apparent trend in the changes in cytokine levels with increasing extract concentration. This re-affirms the observations made in the MTT assays, where increasing concentrations of extracts resulted in higher SDH activity for aqueous but not ethanol extracts.

Tumour Necrosis Factor -alpha (TNF-α, Figure 2), IL-6 ( Figure 3), IL-10 ( Figure 6), the highest levels were observed in PBMCs stimulated with the aqueous extracts of *P. niruri* and the lowest levels observed in stimulations with the ethanol extracts of *M. oleifera*. For IL-1β ( Figure 4), the *P. niruri* aqueous extract stimulation gave the highest cytokine concentrations while the two ethanol extracts yielded comparatively low cytokine concentrations. For IFN-α, the *P. niruri* aqueous extract showed the highest levels ( Figure 5) while for IFN-γ, the two *P. niruri* extracts showed the highest levels ( Figure 7).

## Discussion

Cytokines play an essential role during chronic HBV infection as both innate and adaptive immune cells secret cytokines that are involved in the control of the virus ^30^. The hepatitis B virus is noncytopathic, and infections caused by this virus significantly influence cytokine-mediated immune responses ^7, 31, 32^. For some disorders, immunomodulation utilizing medicinal plants (or their components) can offer alternatives to the standard treatment ^33^. Extracts from various plants have been shown to exhibit immunomodulatory and other relevant activities for the control of both infectious and immune-related noncommunicable diseases. We therefore investigated the effect of *M. oleifera* and *P. niruri* aqueous and ethanol extracts on the activation status of immune cells during the early phase of chronic HBV infection. We measured the levels of the cytokines TNF- α, IL-6, IL-1β, IFN- α, IL-10, and IFN- γ following stimulation of PBMCs from chronic HBV-infected persons and compared them with those of similarly stimulated PBMCs from uninfected persons. These cytokines were selected because they are directly or indirectly involved in the complex network of processes that result in inhibition of HBV replication as well as mediate a non-cytolytic clearance of the virus during acute infections.

Results from this study showed that even at doses as high as 800 μg/ml, the aqueous and ethanol extracts of the two plants were not cytotoxic to human PBMCs ( Figure 1) as they effectively catalyzed the reduction of the MTT dye to formazan. The increase in enzymatic activity of mitochondrial SDH would be a direct effect of the increased metabolic activity or proliferation following treatment with the plant extracts ^29, 34^. Using the Trypan blue exclusion method, it was observed that extracts from both plants, especially the aqueous extracts, induced proliferation in human PBMCs. Previous reports indicate that both the aqueous and ethanol extract of *P. niruri* and *M. oleifera* induced the proliferation of human PBMCs ^23, 35, 36^, which implies that the extracts can alter the immune system. Ethanol is known to cause the dysfunction of the mitochondrion by inducing mitochondrial swelling ^37^. Succinate dehydrogenase is a mitochondrial enzyme, and therefore the action of ethanol on the mitochondrion can affect its function. In our experiments, trace amounts of ethanol may account for the decrease in SDH activity with increasing extract concentration for the ethanol extracts. This observation may also account for the higher SDH activities observed in the ethanol extracts at lower concentrations when the residual ethanol is expected to be diluted out ( Figure 1). Toxicological studies have also shown that both plant extracts are not harmful ^38, 39^.

Phytochemicals such as saponins, flavonoids, alkaloids, tannins, and phenols as identified in both plant extracts are already known to be present in these plants ( Table 1) ^40, 41^, and are known to have immunomodulatory properties ^42, 43^. Quantification of the various phytochemicals revealed that the aqueous extracts were rich in alkaloids, flavonoids, and saponins. This was consistent with previous work, which revealed that aqueous extracts of *M. oleifera* contain``` more phytochemical constituents than ethanol extracts ^44^. Also, Kasolo and colleagues showed that when *M. oleifera* root peel extracts were screened for phytochemicals using aqueous and ethanolic solvents, water extracted more phytochemicals than ethanol, indicating that water is a good solvent for the extraction of leaves ^45^. The ethanol extracts were rich in tannins and phenolic acids, as ethanol is the preferred solvent for the extraction of tannins and phenolic acids because of its ability to dissolve both polar and nonpolar compounds ^46^.

The pathophysiology of HBV has been linked to cytokines, providing evidence that immune-modulating treatment can be effective ^30^. Our results showed that the extracts induced the expression of IL-6, IL-1β, IFN-γ, IL-10, TNF-α, and IFN-α in both the healthy and HBV-infected persons. *Phyllanthus niruri* extract stimulation has been found to enhance the secretion of TNF-α during chronic HBV infection ^47^. Similar results were seen in this study, where there was a high expression of TNF-α. *Moringa oleifera* has been found to stimulate the release of pro-inflammatory cytokines IFN-γ and TNF-α ^48^. Also, *P. niruri* extract administration to mice increased the release of IFN-γ ^49^. In the study reported here, the extracts also increased the release of IFN-γ which is associated with the clearance of the virus during chronic hepatitis B infection by inhibiting HBV gene expression and replication ^50^.

Up to 90 % of the HBV entry into hepatocytes can be inhibited by IL-6 by regulating the expression of sodium taurocholate co-transporting polypeptide (NTCP), which significantly reduces the release of cccDNA and HBsAg ^51^. The high induction of IL-6, by our extracts, suggests that these extracts may aid in the clearance of the virus during chronic hepatitis B infection. Interferon alpha is an essential cytokine during hepatitis B infection. This cytokine is known to inhibit the replication of the virus at several points in the life cycle of the virus ^9, 10, 52^. Synthetic forms of IFN-α have been approved for the treatment of chronic hepatitis B infection. During HBV infection, the host immune system is unable to release type I or III IFN ^53^. Therefore, the ability of the extracts to induce IFN-α expression could aid in treating chronic HBV infection.

PBMCs from the uninfected group were however more responsive than PBMCs from the infected group. This may be because PBMCs from HBV-infected persons have reduced responsiveness to stimulation, and this was consistently shown for all stimulants including the PHA-positive control. This phenomenon is likely associated with immune exhaustion, during the chronic phase of hepatitis B infection, where the immune cells become compromised and begin to express increasing levels of inhibitory markers like PD-1, CTLA-4, and TIM-3 on T cells that prevent the release of these antiviral cytokines ^46, 54^. There could also be an upregulation in the expression of regulatory cytokines like IL-10 and TGF-β that will dampen the relevant pro-inflammatory response which is required to suppress viral replication ^54– 56^. The reduced cytokine expressed could also in part be attributable to a dysfunctional antigen presentation mechanism as is common with chronic viral infections ^57^.

According to work done by Ribeiro and colleagues, there are higher levels of IL-10, TNF-α, IL-2, and IL-6 in patients with acute HBV compared to patients with chronic HBV and healthy individuals ^58^. These cytokines are believed to aid clearance of the virus from hepatocytes and thus prevent infection progression to the chronic forms. The seeming reduction in responsiveness of PBMCs from early chronically HBV-infected persons, relative to healthy controls, could therefore be a contributing factor to why these patients enter chronicity, due to a reduced viral clearance capacity. It is already established that as the chronic state advances, increasing viral load induces a cytokine secretion profile whose side effects result in the disease pathophysiology ^59^.

Even though this study was not designed to establish links between specific plant metabolites and cytokine expression, a number of the plant metabolite classes found in our extracts have been documented to have immunomodulatory properties ^60– 62^. The aqueous extracts of both plants had a high content of alkaloids, saponins, and flavonoids while the ethanol extracts had relatively higher levels of tannins and phenols. We can only speculate that these differences in the abundance of the different metabolites could influence their observed stimulatory activities against PBMCs. Alkaloids containing bis-isoquinoline nuclei like berbamine, tetradrine, and daurince are, for example, known to have significant immunomodulatory properties ^63^. Flavonoids are known to possess significant immunomodulatory properties. These compounds have been reported to enhance cytokine production, modulate immune cell activation, and inhibit viral replication ^64^. Some subclasses of flavonoids have been found to inhibit HBV entry into the hepatocytes ^65^. Also, quercetin is one of many flavonoids that have been found to modulate the generation of Th-1 and Th-2 derived cytokines and have antiviral and anticancer properties ^66^. This might have accounted for the high levels of cytokine expression by the aqueous extracts compared to the ethanol extracts and for the high cytokine levels elicited by the aqueous extract of *P. niruri*, which had the highest content of alkaloids and flavonoids. Tannins, another class of polyphenols, are known for their antiviral and immunomodulatory effects. Tannins such as corilagin have been identified in *Phyllanthus* species and mediate the production of cytokines such as TNF-α, IL-6, and IFN-γ, which are crucial for antiviral immunity ^67, 68^. They are also known to restore immune competence in HBV-infected patients ^47^. Additionally, tannins have hepatoprotective effects, reducing oxidative stress and inflammation in hepatotoxic models, which may contribute to limiting HBV-associated liver damage ^22, 24^.

While the results demonstrate the immunomodulatory potential of extracts from *P. niruri* and *M. oleifera*, some challenges must be addressed before they can be used in clinical settings. A crucial factor to consider is the bioavailability of the active ingredients, such as flavonoids which generally have very low bioavailability and limited systemic absorption due to limited solubility in aqueous environments ^69, 70^. However, this can be improved by the presence of other compound classes such as alkaloids, carotenoids, and vitamins, which are mostly water-soluble ^71, 72^. Standardized dosing procedures are also necessary to guarantee consistent therapeutic results because of the variation in phytochemical concentration between extract batches. To establish a good safety profile, additional research is also necessary to determine any potential adverse effects, especially at higher dosages. Translating these discoveries into effective treatments for chronic HBV will require addressing these issues through advanced formulation procedures and thorough clinical testing.

While this study presents evidence for the immunomodulatory potential of *M. oleifera* and *P. niruri* extracts, these limitations should be kept in mind when interpreting the results. First, we did not fractionate the extracts to identify the specific bioactive compounds responsible for the observed cytokine induction. Therefore, we can only speculate about the contribution of the various phytochemical classes we measured.

Secondly, while we observed a consistent responsiveness of PBMCs from chronic HBV patients, we did not confirm the cause of this immune impairment, such as by evaluating the expression of exhaustion markers (e.g., PD-1, CTLA-4). Consequently, our explanation for this finding remains an inference based on established literature.

Finally, our *in vitro* model using PBMCs, while informative for immunomodulation, cannot directly assess the extracts' ability to inhibit HBV replication or reduce viral load within hepatocytes. The therapeutic potential suggested by cytokine induction needs validation in hepatocyte-based models or in vivo systems. Future studies addressing these limitations will be crucial to fully understanding the mechanism of action and therapeutic promise of these plant extracts. 

In summary, the findings from this study add to the literature on the potential of plant extracts as potential anti-HBV agents and the therapeutic properties of *Phyllanthus niruri* and *Moringa oleifera* extracts may in part be through an immunomodulatory effect. The two plant extracts were largely non-toxic as indicated by data from the MTT assay and induced the expression of HBV-limiting cytokines. The study also highlights possible differences between immune profiles of HBV-infected persons in the early stages of chronicity and those in an advanced stage of chronicity.

## Ethics and consent

Ethical approval was obtained from both the Institutional Review Board (IRB, Federalwide Assurance number FWAA00001824) of NMIMR (NMIMR-IRB/046/19-20, original approval date: 8 ^th^ January 2020) and the Ethics Review Committee of the Korle-Bu Teaching Hospital, Accra (KBTH-IRB/00024/2020, original approval date: 27 ^th^ May 2020). Archived cells used in this study were obtained from study participants who gave written informed consent and willingly agreed to be part of the study. All experiments were conducted following the relevant guidelines and regulations, including the principles of the Belmont Report and the Declaration of Helsinki.

## Data Availability

All relevant data have been presented within the paper. The raw data that were analyzed in this study as well as extended data have been deposited in the OSF online data repository, accessible at https://osf.io/rx73v/overview: Immunomodulatory effect of Moringa oleifera and Phyllanthus niruri extracts on anti-HBV cytokine production by human peripheral blood mononuclear cells (DOI 10.17605/OSF.IO/RX73V) ^73^ Data are available under the terms of the Creative Commons Attribution 4.0 International license (CC-BY 4.0).
