# Supplementary material for: Immunomodulatory effect of Moringa oleifera and Phyllanthus niruri extracts on anti-HBV cytokine production by human peripheral blood mononuclear cells
Source: Open Res Eur. 2025 Dec 4;5:149. Originally published 2025 May 28. [Version 3] doi: 10.12688/openreseurope.20378.3 (PMC12596560; doi:10.12688/openreseurope.20378.3)
Supplement: Peer review history

# Reviewer report: Reviewer response for version 2

**DOI:** 10.21956/openreseurope.23475.r62876

**Published:** 2025-11-24

**Authors:** Sujogya Kumar Panda

**Affiliations:** 1 Siksha ‘O’ Anusandhan (Deemed to be University), Bhubaneswar, Odisha, India

The revised version of the MS can be acceptable.

Is the study design appropriate and does the work have academic merit?

Partly

Is the work clearly and accurately presented and does it cite the current literature?

Yes

If applicable, is the statistical analysis and its interpretation appropriate?

I cannot comment. A qualified statistician is required.

Are all the source data underlying the results available to ensure full reproducibility?

Partly

Are the conclusions drawn adequately supported by the results?

Yes

Are sufficient details of methods and analysis provided to allow replication by others?

Partly

Reviewer Expertise:

NA

I confirm that I have read this submission and believe that I have an appropriate level of expertise to confirm that it is of an acceptable scientific standard.

# Reviewer report: Reviewer response for version 1

**DOI:** 10.21956/openreseurope.22055.r60858

**Published:** 2025-11-08

**Authors:** Michael Adinortey

**Affiliations:** 1 University of Cape Coast, Cape Coast, Ghana

**Summary**

 Information in literature reports that chronic hepatitis B remains a major contributor to cirrhosis and liver cancer worldwide. Current treatment options such as pegylated interferons and nucleoside analogues provide only partial viral control and often cause undesirable side effects, highlighting the need for safer and more effective therapeutic alternatives. This study assessed the aqueous and ethanolic leaf extracts of Moringa oleifera and Phyllanthus niruri on peripheral blood mononuclear cells (PBMCs) sourced from both infected and uninfected individuals. Cytokine levels were quantified using a Luminex multiplex assay, while cell viability and toxicity were evaluated through the MTT assay. Both plant extracts were found to be non-cytotoxic and enhanced cell metabolism. They promoted the production of immune mediators such as IL‑6, IFN‑γ, TNF‑α, IL‑1β, IL‑10 and IFN‑α, which are vital in restricting HBV replication. Nevertheless, cytokine release was generally diminished in cells from infected persons, suggesting immune suppression linked to chronic infection. Overall, the findings demonstrate that the extracts of M. oleifera and P. niruri act as safe immunomodulators capable of encouraging antiviral cytokine responses, potentially aiding immune recovery and viral control. Implicitly the extracts could serve as natural adjunct therapies for managing chronic hepatitis B and also restore immune balance.

**Relevance of work**

 This study is very important as it addresses the global challenge of chronic hepatitis B, a major cause of cirrhosis and liver cancer. Current antiviral therapies often have limited efficacy and undesirable side effects, underscoring the need for safer alternatives. In this article, the evaluation of *Moringa oleifera* and *Phyllanthus niruri* extracts demonstrated non-cytotoxic effects and induced antiviral cytokines such as IL‑6, TNF‑α, and IFN‑γ, which are essential in controlling HBV replication. These findings highlight the potential of plant-based immunomodulators as complementary therapies for enhancing immune response and managing chronic HBV infection.

**Abstract**

 Though the abstract section is well written, there appears to be a high percentage similarity index for that section as evidenced in the Turnitin similarity index results. Authors are advised to re-write that section again to ensure originality.

**Introduction**

 The rationale could be worked on by focusing on the ethnopharmacological relevance linked to HPV and the specific part of plant used. If indigenous people use these plants as decoctions, tincture or in any other form, then authors should state and link it to the choice of solvents used in extraction process.

 Please reframe the sentence “Preventing the progression of the disease to these complicated forms is the major therapeutic goal for persons with chronic HBV infection” as “The main therapeutic goal for individuals with chronic HBV infection is to prevent progression to these complicated forms". Authors are advised to include literature information on specific compounds present in *Moringa oleifera* and *Phyllanthus niruri* leaves. The inclusion would allow for an inference to be drawn under discussion section regarding what in the plant extracts may be contributing to the immunomodulatory effects making the manuscript informative and useful applications.

**Methodology**

 Leaves general dry between 5-7 days under shade. Authors should check their protocol and observation made and present the correct statement under “preparation of extract” section. For Quantitative determination of phytochemicals, authors are advised to cite the specific method (s) used for quantitative analysis of phenols, tannins, flavonoids, alkaloids, and saponins. Authors are also advised to justify the criteria for selection of blood samples in the discussion to strengthen its medicinal relevance.

**Results**

 In presenting data for quantitively phytochemical analysis as shown on table 1, It is advisable to state the results by comparing it with a specific standard used. Authors are advised to specify the specific standard used for each assay.  The number of replications must be indicated. (n = 2 or more) and express concentrations appropriately e.g. mg gallic acid equivalent (GAE)/g extract for phenols; mg diosgenin equivalent (DE)/g extract for saponins etc.

**Discussion**

 The statement “Even though this study was not designed to establish links between specific plant metabolites and cytokine expression, a number of the plant metabolite classes found in our extracts have been documented to have immunomodulatory properties [60–62]……. Additionally, tannins have hepatoprotective effects, reducing oxidative stress and inflammation in hepatotoxic models, which may contribute to limiting HBV-associated liver damage [24,69]” page 12 of 17 appears to be speculative as authors didn’t link observed immunomodulatory properties to any specific reported compound (s) found in the leaves of plants studied. I suggest authors check literature on compounds present in these plants and connect observed pharmacological activity to it.

 Please reframe the statement “ In summary, the findings from this study add to the literature on the potential of plant extracts as potential anti-HBV agents

 and the therapeutic properties of Phyllanthus niruri….” Page 12 of 17

Is the study design appropriate and does the work have academic merit?

Yes

Is the work clearly and accurately presented and does it cite the current literature?

Yes

If applicable, is the statistical analysis and its interpretation appropriate?

Yes

Are all the source data underlying the results available to ensure full reproducibility?

Yes

Are the conclusions drawn adequately supported by the results?

Yes

Are sufficient details of methods and analysis provided to allow replication by others?

Yes

Reviewer Expertise:

Pharmacology, Medicinal Biochemistry, Natural Products, Clinical Biochemistry, Computational Chemistry

I confirm that I have read this submission and believe that I have an appropriate level of expertise to confirm that it is of an acceptable scientific standard.

# Response

**Published:** 2025-11-16

**Authors:** Kwadwo Kusi

**Affiliations:** Immunology, Noguchi Memorial Institute for medical Research, University of Ghana, Legon, Accra, Ghana

**Relevance of work**

 This study is very important as it addresses the global challenge of chronic hepatitis B, a major cause of cirrhosis and liver cancer. Current antiviral therapies often have limited efficacy and undesirable side effects, underscoring the need for safer alternatives. In this article, the evaluation of *Moringa oleifera* and *Phyllanthus niruri* extracts demonstrated non-cytotoxic effects and induced antiviral cytokines such as IL‑6, TNF‑α, and IFN‑γ, which are essential in controlling HBV replication. These findings highlight the potential of plant-based immunomodulators as complementary therapies for enhancing immune response and managing chronic HBV infection.

**Abstract**

**Comment** Though the abstract section is well written, there appears to be a high percentage similarity index for that section as evidenced in the Turnitin similarity index results. Authors are advised to re-write that section again to ensure originality.  

**Response: **We have checked, and the similarity is mostly with earlier versions of this same paper in the submissions system, as well as the thesis submitted by the student who conducted this work, from which this paper is derived. In our opinion, these are not significant basis to concluded that the abstract is plagiarized.

**Introduction** **Comment**

 The rationale could be worked on by focusing on the ethnopharmacological relevance linked to HPV and the specific part of plant used. If indigenous people use these plants as decoctions, tincture or in any other form, then authors should state and link it to the choice of solvents used in extraction process.

**Response: **This has already been addressed in the introduction and the methodology; however, a sentence has been added to the introduction to meet the reviewer's requirement (Page 7 of 24).  

**Comment**

 Please reframe the sentence “Preventing the progression of the disease to these complicated forms is the major therapeutic goal for persons with chronic HBV infection” as “The main therapeutic goal for individuals with chronic HBV infection is to prevent progression to these complicated forms". Authors are advised to include literature information on specific compounds present in *Moringa oleifera* and *Phyllanthus niruri* leaves. The inclusion would allow for an inference to be drawn under discussion section regarding what in the plant extracts may be contributing to the immunomodulatory effects making the manuscript informative and useful applications.  

**Response: **The statement has been rephrased (page 6 of 24). Literature information on the specific compounds present in the extracts has been added to the introduction section of the manuscript (Page 7 of 24).  

**Methodology** **comment**

 Leaves general dry between 5-7 days under shade. Authors should check their protocol and observation made and present the correct statement under “preparation of extract” section. **Response**: We have appropriately revised this to reflect specifically what was done, as below Revised version: The leaves of both plants were washed and shade-dried for seven (7) days (page 8 of 24).  

**Comment** For Quantitative determination of phytochemicals, authors are advised to cite the specific method (s) used for quantitative analysis of phenols, tannins, flavonoids, alkaloids, and saponins.

**Response:** The specific methods used for the quantification has been cited in the introduction of that section (page 8 of 24).    

**Comment** Authors are also advised to justify the criteria for selection of blood samples in the discussion to strengthen its medicinal relevance.  

**Response:** This comment has been addressed in the discussion section (page 16 of 24) by the addition of the paragraph below *“PBMCs were obtained from individuals who* *were in* *the early chronic phase of HBV infection* *,* *who had not yet started antiviral treatment* *. This cohort was* *chosen to examine the* *immunomodulatory potential* *of the plant extracts on the immune system before it is potentially altered by long-term high viral load, advanced liver disease, or the effects of pharmaceutical interventions, thereby providing a clearer insight into their therapeutic relevance for early intervention.”*

**Results** **comment**

 In presenting data for quantitively phytochemical analysis as shown on table 1, It is advisable to state the results by comparing it with a specific standard used. Authors are advised to specify the specific standard used for each assay.  The number of replications must be indicated. (n = 2 or more) and express concentrations appropriately e.g. mg gallic acid equivalent (GAE)/g extract for phenols; mg diosgenin equivalent (DE)/g extract for saponins etc.  

**Response:** All corrections have been made to Table 1 (Page 11 of 24).  

**Discussion** **Comment**

 The statement “Even though this study was not designed to establish links between specific plant metabolites and cytokine expression, a number of the plant metabolite classes found in our extracts have been documented to have immunomodulatory properties [60–62]……. Additionally, tannins have hepatoprotective effects, reducing oxidative stress and inflammation in hepatotoxic models, which may contribute to limiting HBV-associated liver damage [24,69]” page 12 of 17 appears to be speculative as authors didn’t link observed immunomodulatory properties to any specific reported compound (s) found in the leaves of plants studied. I suggest authors check literature on compounds present in these plants and connect observed pharmacological activity to it.  

**Response: **That section of the discussion has been rephrased and linked to the metabolites found in the plant extracts (Page 18 of 24).

**Comment**

 Please reframe the statement “In summary, the findings from this study add to the literature on the potential of plant extracts as potential anti-HBV agents

 and the therapeutic properties of *Phyllanthus niruri*….” Page 12 of 17  

**Response: **The statement has been accordingly rephrased (Page 19 of 24).

# Reviewer report: Reviewer response for version 1

**DOI:** 10.21956/openreseurope.22055.r60857

**Published:** 2025-10-08

**Authors:** Edward Maina

**Affiliations:** 1 Kenya Medical Research Institute, Nairobi, Kenya

The author presents an important piece of data that may significantly inform drugs discovery targets. These data provide primary findings that should be explored further to inform management of HBV. However, the author should be able to explain why PBMCs from the uninfected group were more responsive than PBMCs from the infected group. If it is because PBMCs from the HBV-infected persons have reduced responsiveness to stimulation, as suggested, was this aspect confirmed? The author should also clearly explain the limitation of the study to inform interpretation of the results in the context of limitations therein. Overall, if concerns raised herein are addressed, the article presents indexable data that will benefit the wider readership and inform further studies.

Is the study design appropriate and does the work have academic merit?

Yes

Is the work clearly and accurately presented and does it cite the current literature?

Yes

If applicable, is the statistical analysis and its interpretation appropriate?

Yes

Are all the source data underlying the results available to ensure full reproducibility?

Yes

Are the conclusions drawn adequately supported by the results?

Yes

Are sufficient details of methods and analysis provided to allow replication by others?

Yes

Reviewer Expertise:

Infectious diseases and Immunology

I confirm that I have read this submission and believe that I have an appropriate level of expertise to confirm that it is of an acceptable scientific standard.

# Response

**Published:** 2025-10-21

**Authors:** Kwadwo Kusi

**Affiliations:** Immunology, Noguchi Memorial Institute for medical Research, University of Ghana, Legon, Accra, Ghana

- However, the author should be able to explain why PBMCs from the uninfected group were more responsive than PBMCs from the infected group. If it is because PBMCs from the HBV-infected persons have reduced responsiveness to stimulation, as suggested, was this aspect confirmed

**Response:** This has already been explained in the discussion section. However, this study did not confirm the cause of this responsiveness, but the pattern observed provides a strong rationale for such investigations in the future.  

- The author should also clearly explain the limitation of the study to inform interpretation of the results in the context of limitations therein

**Response:** We thank the reviewer for this important suggestion to enhance the clarity and rigor of our manuscript. In response, while there was a section in the earlier submission that described the study limitations, we have revised this section within the discussion to reflect our earlier identified limitations and additional ones raised by reviewers.

# Reviewer report: Reviewer response for version 1

**DOI:** 10.21956/openreseurope.22055.r55965

**Published:** 2025-09-05

**Authors:** Sujogya Kumar Panda

**Affiliations:** 1 Siksha ‘O’ Anusandhan (Deemed to be University), Bhubaneswar, Odisha, India

- *Consider elaborating on the rationale for selecting these specific plants.* Were they chosen based on ethnobotanical relevance, prior antiviral evidence, or known immunomodulatory potential?
- *Why was 100 μl of DMSO used as the negative control for the MTT assay, while water was used in other cases?* Ethanol extracts are indeed difficult to dissolve in water. The choice of solvents is unclear—why were only aqueous and ethanolic extracts used, without including a hydrophobic solvent for comparison?
- *Were any controls other than PHA used to benchmark cytokine induction?* For example, known mitogens like LPS could have provided a more robust comparison. Including such controls would strengthen the interpretation of immunostimulatory effects.
- *The discussion could be expanded to explore the implications of reduced cytokine responsiveness in HBV-infected PBMCs.* Could this be linked to T-cell exhaustion, immune senescence, or regulatory cell dominance?
- *The major drawback is the lack of characterization of active compounds.* The current phytochemical analysis is preliminary and does not add substantial scientific value. It is recommended to isolate active constituents or at least perform HPTLC fingerprinting to enable future researchers to build upon this work.

Is the study design appropriate and does the work have academic merit?

Partly

Is the work clearly and accurately presented and does it cite the current literature?

Yes

If applicable, is the statistical analysis and its interpretation appropriate?

I cannot comment. A qualified statistician is required.

Are all the source data underlying the results available to ensure full reproducibility?

Partly

Are the conclusions drawn adequately supported by the results?

Yes

Are sufficient details of methods and analysis provided to allow replication by others?

Partly

Reviewer Expertise:

Natural products

I confirm that I have read this submission and believe that I have an appropriate level of expertise to state that I do not consider it to be of an acceptable scientific standard, for reasons outlined above.

# Response

**Published:** 2025-10-21

**Authors:** Kwadwo Kusi

**Affiliations:** Immunology, Noguchi Memorial Institute for medical Research, University of Ghana, Legon, Accra, Ghana

- *Consider elaborating on the rationale for selecting these specific plants.* Were they chosen based on ethnobotanical relevance, prior antiviral evidence, or known immunomodulatory potential?

**Response:** The selection of *Moringa oleifera* and *Phyllanthus niruri* was indeed deliberate and based on three key factors (ethnobotanical relevance, immunomodulatory properties, and antiviral properties), which were briefly outlined in the introduction. Additional information on this has been added (lines108-111).  

- *Why was 100 μl of DMSO used as the negative control for the MTT assay, while water was used in other cases?* Ethanol extracts are indeed difficult to dissolve in water. The choice of solvents is unclear—why were only aqueous and ethanolic extracts used, without including a hydrophobic solvent for comparison?

**Response**: The 100 μl of DMSO was not used as a negative control but as a solubilization agent following the standard MTT assay protocol. For clarity, the MTT assay section of the methodology has been updated. The reasons for using water and ethanol for preparation of the extracts under the methodology section has now been justified in the methods section.  

- *Were any controls other than PHA used to benchmark cytokine induction?* For example, known mitogens like LPS could have provided a more robust comparison. Including such controls would strengthen the interpretation of immunostimulatory effects.

**Response: ** The primary goal of our cell stimulation assay was to evaluate the potential of plant extracts to activate lymphocytes within the PBMC population, as T cells are central to the adaptive immune response against HBV. With this specific aim, we selected Phytohaemagglutinin (PHA) as our positive control. PHA is a well-established and potent T-cell mitogen. Its robust and predictable induction of a broad range of cytokines from T cells provided a clear and relevant benchmark for the immunostimulatory capacity of our plant extracts on the adaptive immune arm. PHA can however also indirectly activate innate immune cells like macrophages and dendritic cells through secondary mechanisms by inducing cytokines and other signaling molecules from activated lymphocytes [ref 1]. We agree that investigating the effects on innate immune cells is also of interest.   While Lipopolysaccharide (LPS) is indeed a classic mitogen, it primarily activates the innate immune system by targeting monocytes/macrophages and B cells via Toll-like receptor 4 (TLR4). Since our study's focus was on cytokine production with relevance to T-cell-mediated viral control, PHA was the more targeted and appropriate choice.  

- *The discussion could be expanded to explore the implications of reduced cytokine responsiveness in HBV-infected PBMCs.* Could this be linked to T-cell exhaustion, immune senescence, or regulatory cell dominance?

**Response:** This comment had already been addressed in the discussion in lines 438-448.  

- *The major drawback is the lack of characterization of active compounds.* The current phytochemical analysis is preliminary and does not add substantial scientific value. It is recommended to isolate active constituents or at least perform HPLC fingerprinting to enable future researchers to build upon this work.

**Response:** HPLC fingerprinting was performed, and the methods and outcomes have now been added to the methods and results sections.  The fingerprint chromatograms have been added to the data repository that accompanies the manuscript.  

**Reference** 1. Lomakova YD, Londregan J, Maslanka J, Goldman N, Somerville J, Riggs JE. PHA eludes macrophage suppression to activate CD8+ T cells. Immunobiology. 2019;224:94–101. https://doi.org/10.1016/j.imbio.2018.10.004.
